# To the Medical Profession

**Published:** 1860-01

**Authors:** A. Hard

**Affiliations:** Aurora, Ill.


### TO THE MEDICAL PROFESSION.

At the Annual Meeting of the *Illinois State Medical Society*, held at Decatur on the first Tuesday in June last, the undersigned was appointed a special committee on the Medical uses of *Veratrum viride.*

Being desirous of gaining all the information within reach, bearing upon the subject, so as to make as full and satisfactory a report as possible, I take the liberty of addressing you the following questions, and respectfully solicit an early answer.

1. Have you made use of *veratrum viride* in your practice? If you have:

2. In what form do you use it; (if the tincture; whose preparation?) and in what dose?

3. What are its effects?

4. In your opinion, what is its *modus operandi?*

5. What value do you attach to it as a remedial agent?

6. In what diseases have you found it most useful?

Give any other information upon the subject you may possess, and address A. Hard, M. D., Aurora, Kane County, Ill., as early as the first of March, 1860.

Yours, &c.,

*Aurora, Ill., Nov.* 28, 1859. A. HARD.

